# Factors influencing the use of postoperative bilevel positive airway pressure (BiPAP) in patients undergoing adult cardiac surgery: A retrospective cohort study

**DOI:** 10.1002/hsr2.873

**Published:** 2022-10-03

**Authors:** Syed S. Ahmed, Muhammad S. Yousuf, Khalid Samad, Hameed Ullah, Khalid M. Siddiqui

**Affiliations:** ^1^ Department of Anaesthesiology Aga Khan University Karachi Pakistan

**Keywords:** acute respiratory failure (ARF), BiPAP, cardiac surgery, mechanical ventilation, noninvasive ventilation (NIV)

## Abstract

**BAckground and Aims:**

Respiratory complications are one of the biggest challenges following cardiac surgery, which can lead to hypoxia and acute respiratory failure (ARF). The aim of this study to identify the factors led to BiPAP application for postoperative respiratory complications and its effectiveness as the main outcome measures after cardiac surgery.

**Methods:**

It was a retrospective cohort study with consecutive sampling technique. A total of 335 postcardiac surgery patients medical record was reviewed who were underwent for surgery from November 1, 2018 to November 30, 2019. 265 patients were finalized for the recruitment, five patients were excluded before the final analysis. Data of 260 patients were analyzed for compiling of results.

**Results:**

The mean age was 59 years. 196 (75.4%) patients were males and females were 64 (24.6%). Mean weight was 72 kg and mean body mass index (BMI) 26.67 kg/m^2^_._ BiPAP application was in 38 (14.6%) patients and significantly high in with high BMI, (*p* < 0.05). There are significant associations of BiPAP application patients with COPD (*p* < 0.05). Patients with positive fluid balance, cardiac dysfunction, and required inotropic support were significantly associated with BiPAP need (*p* < 0.05), respectively.

**Conclusion:**

BiPAP is effective to treat ARF and other respiratory complications after adult cardiac surgeries. High BMI, atelectasis, and pneumonia are also the independent factors causing ARF. BiPAP can be a successful tool for preventing the adverse effects of postoperative pulmonary complications after cardiac surgery.

## 1 INTRODUCTION

Postcardiac surgeries pulmonary problems are always matter of worries for both cardiac surgeons and anesthesiologists. These complications could have variety of mild respiratory impairment to acute respiratory distress syndrome (ARDS). Atelectasis is one of the known primary trigger of many complications, and further may lead to hypoxemia and pneumonia. Respectively one of these issues raises the risk of morbidity and mortality.^1^

Postoperative risk of respiratory complications is increased by anesthesia, pain of sternotomy cardiopulmonary bypass, thoracotomy, diaphragm malfunction, fluid overload, major transfusions, and the patient's pre‐existing condition. These problems are linked to a longer stay in the hospital and a lower chance of recovery.^2^

Despite advances in perioperative care, after cardiac surgery respiratory failure remains a common complication after cardiopulmonary bypass (CPB). It further leads to mortality and morbidity. To minimize pulmonary function impairment, various techniques have been developed, including perioperative mechanical ventilation (MV), restrictive transfusion, technological modifications of CPB, and drug administration, such as steroids and aprotinin.^3^, ^4^

Application of noninvasive ventilation (NIV) like BiPAP, using face or nose masks has reduced the necessity of endotracheal intubation. It has been recognized that BiPAP can prevent atelectasis and postoperative pneumonia, it also has beneficial effects in postoperative phase of cardiac surgery to prevent other pulmonary complications.

The main purpose of this study to see the factors led to BiPAP application for postoperative respiratory complications and observed its effectiveness as main outcome measures in patients after cardiac surgery.

### 1.1 Objectives

The main objectives of this study to see the factors led to BiPAP application and its effectiveness to manage postoperative respiratory complications as main outcome measures in patients after cardiac surgery.

## 2 MATERIALS AND METHODS

### 2.1 Design

**Figure 1:**
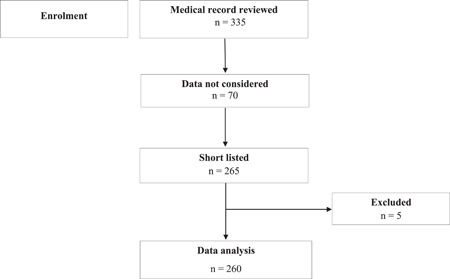
Flowchart of patients who had cardiac surgery

It was retrospective cohort study with consecutive sampling technique. The consent was not required as there was no direct involvement and questioning to patients. General and thoracic surgery patients were excluded. All data were collected from patient's medical record. Total of 335 patients' medical record was reviewed who were undergone for cardiac surgery. 70 patients' data were not considered due to missing information. Finally, 265 patients were finalized for the recruitment. Five patients were excluded before final analysis, in where three patients had postoperative cerebrovascular complication and two patients were died within 48 h in postoperative phase. Therefore, data of 260 patients were analyzed for compiling of results (Figure 1).

The data was collected on pre‐designed data collecting form for the parameters collected in the preoperative location, induction/operating rooms, and the postoperative events of the CICU after cardiac surgery. The principal reasons for the application of BiPAP, length of stay in the CICU, postoperative complications, and outcome in terms of mortality are also noted in all patients.

### 2.2 Settings

As it was a retrospective cohort study, therefore it was exempted by the Ethical Review Committee of the institution. Patients’ clinical records were reviewed from November 1, 2018 to November 30, 2019.

### 2.3 Participants

All records of the patients with either gender scheduled for cardiac surgery at cardiac operating rooms, then shifted in coronary care unit (CICU) after surgery were included.

### 2.4 Main outcomes measures

The main outcome measures of this study to identify the factors led to BiPAP application and its effectiveness for postoperative respiratory complications in patients after cardiac surgery.

### 2.5 Statistical analysis

All statistical analyses were carried out using version 21 of the statistical packages for social science (SPSS Inc.). Median and 25th–75th percentile was computed for quantitative variables and analyzed by Mann–Whitney *U* test. Whereas qualitative variables were reported in term of frequency and percentage and analyzed by Chi‐square or Fisher's exact test. For the two‐sided tests, *p*‐value < 0.05 was considered the significant threshold.

## 3 RESULTS

Overall documentation compliance was found 79.10%. In demographic data (Table 1) mean aged was 59 years. 196 (75.4%) patients were males and females were 64 (24.6%). Mean weight was 72 kg and mean BMI 26.67 kg/m^2^ and application of BiPAP was significantly high in obese patients with high BMI, *p*‐value < 0.05.

**Table 1:** Demographic values

Variables	Overall	BiPAP used	BiPAP not used	*p*‐value
	*n* = 260	*n* = 38	*n* = 222	
Age (years)^a^	59 (52–65)	60.5 (56–67.75)	59 (50.2–65)	NS
BMI (kg/m^2^)^a^	26.67 (26.67–30.47)	30.2 (27.31–32.89)	26.29 (24.14–30.24)	<0.01
Gender^b^				
Male	196 (75.4%)	26 (68.4%)	170 (76.6%)	NS
Female	64 (24.6%)	12 (31.6%)	52 (23.4%)	

Abbreviation: BiPAP, bilevel positive airway pressure.

NS (nonsignificant) ≥ 0.05.

^a^ Mann–Whitney *U* test.

^b^ Chi‐square test.

Majority of patients 215 (82.7%) had surgery of coronary artery bypass graft surgery (CABG). BiPAP was applied only in 38 (14.6%) patients. Rest of the other procedure and application of BiPAP are given in Table 2. There are significant association of BiPAP need with chronic obstructive pulmonary disease (COPD) where (*p* < 0.05), details of other co‐morbids are given in Table 3. Additionally, postoperative application of BiPAP was high in patients who were already intubated and operated on emergency basis.

**Table 2:** Details of the procedures

Variables	Overall	BiPAP	BiPAP
	*n* = 260	Used *n* = 38	Not used *n* = 222
Procedure*			
CABG	215 (82.69%)	26 (12.09%)	189 (87.9%)
AVR	16 (6.15%)	4 (25%)	12 (6.9%)
DVR	3 (1.15%)	1(33%)	2 (1.3%)
MVR	9(4.23%)	5 (45.45%)	4 (3.9%)
CABG + AVR	3 (1.15%)	0	3 (1.3%)
CABG + MVR	5 (1.92%)	2 (40%)	5 (2.2%)
AVR + MVR	1 (0.38%)	0	1 (0.4%)
ASD closer	3 (1.15%)	0	3 (1.3%)
Aortic root	1 (0.38%)	0	1 (0.9%)
Surgery			
Others	2 (0.76%)	0	2 (0.4%)

Abbreviation: BiPAP, bilevel positive airway pressure.

* ASD, atrial septal defect; AVR, aortic valve replacement; CABG, coronary artery bypass grafting; DVR, double valve replacement; MVR, mitral valve replacement.

**Table 3:** Details of the comorbid of the patients

Comorbid*	Overall *n* = 260	BiPAP used *n* = 38	BiPAP not used *n* = 222	*p*‐value
CVA^b^	8 (3.1%)	2 (5.3%)	6 (2.7%)	NS
Hypertension^a^	191 (73.5%)	26 (68.4%)	165 (74.3%)	NS
Diabetic mellitus^a^	125 (48.1%)	16 (42.1%)	109 (49.1%)	NS
IHD^a^	50 (19.2%)	8 (21.2%)	42 (18.9%)	NS
AKI^b^	6 (2.3%)	0 (0%)	6 (2.7%)	NS
PVD^b^	2 (0.8%)	0 (0%)	2 (0.9%)	NS
Smoke	29 (11.2%)	1 (2.6%)	28 (12.6%)	NS
CKD^a^	21 (8.1%)	5 (13.2%)	16 (7.2%)	NS
NSTEMI^b^	10 (3.8%)	1 (2.6%)	9 (4.1%)	NS
COPD^b^	8 (3.1%)	6 (15.8%)	2 (0.9%)	<0.001
Asthma^b^	9 (3.5%)	0 (0%)	9 (4.1%)	NS
OSA^b^	10 (3.8%)	2 (5.3%)	8 (3.6%)	NS

*Note*: NS (nonsignificant) ≥ 0.05; data are presented as *n* (%).

Abbreviation: BiPAP, bilevel positive airway pressure.

* AKI, acute kidney injury; CKD, chronic kidney disease; COPD, chronic obstructive pulmonary disease; NSTEMI, non‐ST‐elevation myocardial infarction;OSA, obstructed sleep apnea; PVD, peripheral vascular disease; VA, cerebrovascular accident.

^a^ Chi‐square test.

^b^ Fisher's Exact test.

We have observed some intraoperative variables that could effect on outcome in terms of postoperative BiPAP need. We have found that patients with positive fluid balance, and patients with cardiac dysfunction requiring inotropes were significantly associated with respiratory complications and BiPAP application. There is *p*‐values <0.05 respectively shown in Table 4.

**Table 4:** Intra operative parameters of patients with and without BiPAP (*n* = 260)

Variables	Overall *n* = 260	BiPAP	BiPAP	*p*‐value
		Used *n* = 38	Not used *n* = 222	
Ventilation strategy				
Pre‐pump^c^				
PCV	6	2 (33.3%)	4 (66.7%)	NS
VCV	254	36 (14.2%)	218 (85.8%)	
Post‐pump^c^				
PCV	6	2 (33.3%)	4 (66.7%)	NS
VCV	254	36 (14.2%)	218 (85.8%)	
Duration of surgery^a^	300 (253.7–360)	350 (270–360)	300 (250–360)	NS
Pump time (min)^a^	100 (85–130)	105 (90–135.7)	100 (85–127)	NS
Cross clamp time (min)^a^	65 (48.5–85)	75 (50–97.5)	65 (45.7–85)	NS
Fluid balance^c^				
Positive	152	20 (13.2%)	132 (86.8%)	<0.05
Negative	72	17 (23.6%)	55 (76.4%)	
Equal	36	1 (2.8%)	35 (97.2%)	
Use of inotropes^b^				
On	229	29 (12.7%)	200 (87.3%)	<0.05
Off	31	9 (29%)	22 (71%)	

*Note*: NS, nonsignificant ≥ 0.05; data are presented as median [25–75 percentile] and *n* (%).

Abbreviations: BiPAP, bilevel positive airway pressure; PCV, pressure control ventilation; VCV, volume control ventilation.

^a^ Mann–Whitney *U* test.

^b^ Chi‐square test.

^c^ Fisher's exact test.

We have also monitored triggers and clinical conditions which have leads to BiPAP application postoperatively are shown in Table 5.

**Table 5:** Triggers and clinical conditions of BiPAP application post operatively

Triggers for BiPAP application	Count	Percent
Decrease pO_2_ (<60 mmHg)	21	55.2%
Increase pCO_2_ (>50 mmHg)	29	76.3%
Increase RR (>35 breaths/min)	13	34.2%
SpO_2_ < 88%	27	71.05%
Clinical conditions BiPAP application		
Pulmonary edema	9	23.6%
Labored breathing	4	10.5%
Pleural effusion	2	5.26%
Atelectasis	17	44.7%
Lung collapse	6	15.7%
Pneumonia	7	18.4%

*Note*: Data are presented as *n* (%).

Abbreviations: pO_2_, partial pressure of oxygen; pCO_2_, partial pressure of carbon dioxide; RR, respiratory rate; SpO_2_, oxygen saturation.

## 4 DISCUSSION

Respiratory dysfunction, varies from minor to major, is a known side effect of cardiac surgery. The length of cardiopulmonary by‐pass, dysfunction of diaphragm, major transfusion, postoperative pain, fluid overload, and the patient's pre‐existing comorbidities all lead to the jeopardy of respiratory complications following surgery.^5^, ^6^, ^7^, ^8^, ^9^, ^10^, ^11^ BiPAP as a part of noninvasive ventilation (NIV), has been evaluated in after cardiac surgery to prevent acute respiratory failure ARF.^12^

The severity of these surgical complications might extent from minor pulmonary ailment to acute respiratory distress syndrome (ARDS).^13^, ^14^ Atelectasis is one of the frequent pulmonary complications that can develop following cardiothoracic surgery. The effect of general anesthesia, cardiopulmonary bypass (CPB), gas exchange impairment during surgery, and ceasing lung perfusion, are the major causes of atelectasis.^15^ Each of these complications increases the incidence of morbidity and mortality.

BiPAP as a noninvasive ventilation approach has minimized the need for endotracheal intubation. Several studies have recently shown that BiPAP can improve hypoxemia and reduce atelectasis in patients after extubation following to cardiac surgery.^16^, ^17^, ^18^, ^19^, ^20^

Obese cardiac surgery patients have higher rates of hypoxemia, atelectasis, and respiratory dysfunction, and short‐term use of BiPAP improved pulmonary function and reduced the need for re‐intubation.^21^ In our study we have found that increase in BMI is strongly associated with need of application of BiPAP after cardiac surgery. It also showed improvement in respiratory parameters after application of BiPAP in obese patients.

Patients with high BMI had lower postoperative inspiratory capacity (IC) than those who were of average weight. Obesity reduces lung volumes postoperatively, even if it is mild, and the explanation for the lesser IC is possibly the compressed form of diaphragm and the pressure caused by abdominal adipose tissues, both of which minimize the whole space available for the lungs. As a result, patients with a high BMI are more likely to have reduced pulmonary volumes following surgery. Obese patients have further likely prone to develop atelectasis than normal‐weight patients, and their time in the critical care areas and overall hospital stay is longer.^22^

Another major finding in our study was patients with existing chronic obstructive pulmonary disease (COPD) have higher need of BiPAP after cardiac surgery. After surgery, COPD and ischemic heart disease are considered independent risk factors for mortality and severe cardiopulmonary complications. Many surgical candidates have isolated or combined risk factors for coronary artery disease, heart failure, and COPD. Perioperative optimization of these high‐risk patients necessitates a detailed knowledge of the patient's cardiopulmonary diseases, as well as the surgical and anesthetic respiratory implications. Noninvasive ventilation including BiPAP can avoid the re‐intubation and deleterious effects of prolonged mechanical ventilation.^23^ Besides chronic obstructive pulmonary disease (COPD), history of smoking, overall health status, and advance age are important aspects which are linked to higher risk of reduced lung volume following surgery, and the risk amplifies with cardiac surgery.^24^

A study by Lin^25^ and colleagues recognized when compared to patients had not COPD, with geriatric patients along COPD had substantially higher postoperative adverse outcome rates, with a nearly twofold chance of 30‐day mortality. A meta‐analysis by Zhao^26^ and colleagues, patients with COPD have higher risk of getting postoperative morbidities, including stroke, pneumonia, respiratory failure, renal failure, and wound infection. Therefore, extra attentiveness should be taken when patients with COPD are scheduled for cardiac surgery. Therefore, managing pulmonary dysfunction after cardiac surgery is a multistep process that begins before surgery and continues during the operative and postoperative phases. Pulmonary protection measures have evolved over the period with varying degrees of success, and more understanding of available noninvasive ventilation modalities such as BiPAP will lead to better outcomes, which will help to minimize pulmonary dysfunction and improve early outcome and costs following cardiac surgery.^27^

Fluid balance, a potentially adjustable factor, is associated with weaning outcomes. Extravasation of fluid into the extracellular compartment, caused by elevated hydrostatic pressure, is one possible explanation. This extravasation can cause pulmonary edema and a decline in pulmonary function, as well as widen the time spent on mechanical ventilation and it is a well‐known fact that fluid overload reduces the time of mechanical ventilation.^28^, ^29^ Large volume fluid infusion during surgery, clinically presented as pulmonary and peripheral edema. We found that overall accumulated fluid equilibrium was found to be highly associated with the duration of mechanical ventilation. We also found that application of BiPAP cause statistically significant improvement in PaO_2_ within 30 min of application in patients who have develop pulmonary edema especially in those patients in those patients in whom PaO_2_ was less than 60 mmHg.

We have also looked patients for the need of BiPAP with or without inotropic support and found that patients who required inotropic support significantly required mechanical ventilation, and BiPAP were applied for breathing support. That finding supports existing literature, that inotropic support to help in weaning the patients from mechanical ventilation. Routsi and colleagues^30^ have already described weaning‐induced cardiovascular dysfunction in their recent review and conclude that weaning failure is caused or contributed by cardiovascular dysfunction. The cardiovascular dysfunction was confirmed by echocardiography in all patients and weaning failure was associated or exacerbated by cardiovascular dysfunction. Early detection and accurate diagnosis of high‐risk patients with weaning failure of cardiovascular origin are critical, as targeted treatment based on the underlying mechanism can enable the heart to tolerate the pressure of the weaning process more effectively.

Timely change of inspiratory positive airway pressure (IPAP) and expiratory positive airway pressure (EPAP) according to the needs of each patient is an important factor in the success of noninvasive mechanical ventilation. Therefore, the IPAP settings of 12–14 cmH_2_O and EPAP of 6–8 cmH_2_O were used. Since each patient needs a different level of ventilatory assistance, adjustments to the IPAP for sufficient ventilation should be performed by specialized professionals. This individualized modification may explain the discrepancies between noninvasive ventilation studies. The EPAP is adjusted based on factors that benefit alveolar collapse, such as airway flexibility and mechanical changes in the abdomen.

The main limitations of our study are that, it was a retrospective, based on the data of a single center CICU, we did not separate fast‐track recovery protocol patients with conventional endotracheal extubation. Our main focused on the factors which influenced to led BiPAP application after cardiac surgery. A prospective randomized controlled trial may provide more attested results on efficacy of BiPAP application.

## 5 CONCLUSION

In the postoperative period following cardiac surgery, application of BiPAP is considered an efficient and effective to manage respiratory complications. High BMI, atelectasis and pneumonia are the independent factors causing acute respiratory failure after cardiac surgery. BIPAP can be acquired as a useful method for preventing the deleterious repercussions of cardiac surgery on postoperative pulmonary complications.

## AUTHOR CONTRIBUTIONS

**Syed Shabbir Ahmed**: Conceptualization. **Muhammad Saad Yousuf**: Formal analysis; writing – review & editing. **Khalid Samad**: Conceptualization. **Hameed Ullah**: Writing – review & editing. **Khalid Maudood Siddiqui**: Formal analysis; writing – original draft; writing – review & editing.

## CONFLICTS OF INTEREST

The authors declare no conflicts of interest.

## ETHICS STATEMENT

Participant consent is not applicable as the data was required from Patient Medical record. Whereas the study got exemption from Ethical Review Committee of Aga Khan University Hospital. All methods were performed in accordance with the relevant guidelines and regulations. The consent was obtained for all patient record, personally identifiable data.

## TRANSPARENCY STATEMENT

The lead author Khalid Maudood Siddiqui affirms that this manuscript is an honest, accurate, and transparent account of the study being reported; that no important aspects of the study have been omitted; and that any discrepancies from the study as planned (and, if relevant, registered) have been explained.

## Data Availability

The data that support the findings of this study are available on request from the corresponding author. The data are not publicly available due to privacy or ethical restrictions.
